# Novel Immunomodulatory Cytokine Regulates Inflammation, Diabetes, and Obesity to Protect From Diabetic Nephropathy

**DOI:** 10.3389/fphar.2019.00572

**Published:** 2019-05-22

**Authors:** Vikram Sabapathy, Marta E. Stremska, Saleh Mohammad, Rebecca L. Corey, Poonam R. Sharma, Rahul Sharma

**Affiliations:** ^1^Center for Immunity, Inflammation and Regenerative Medicine, Division of Nephrology, Department of Medicine, University of Virginia, Charlottesville, VA, United States; ^2^Department of Microbiology, Immunology, and Cancer Biology, University of Virginia, Charlottesville, VA, United States; ^3^Department of Biomedical Engineering, University of Virginia, Charlottesville, VA, United States

**Keywords:** IL-2, IL-33, diabetes, obesity, nephropathy, inflammation, Treg, ILC2

## Abstract

Obesity-linked (type 2) diabetic nephropathy (T2DN) has become the largest contributor to morbidity and mortality in the modern world. Recent evidences suggest that inflammation may contribute to the pathogenesis of T2DN and T-regulatory cells (Treg) are protective. We developed a novel cytokine (named IL233) bearing IL-2 and IL-33 activities in a single molecule and demonstrated that IL233 promotes Treg and T-helper (Th) 2 immune responses to protect mice from inflammatory acute kidney injury. Here, we investigated whether through a similar enhancement of Treg and inhibition of inflammation, IL233 protects from T2DN in a genetically obese mouse model, when administered either early or late after the onset of diabetes. In the older mice with obesity and microalbuminuria, IL233 treatment reduced hyperglycemia, plasma glycated proteins, and albuminuria. Interestingly, IL233 administered before the onset of microalbuminuria not only strongly inhibited the progression of T2DN and reversed diabetes as indicated by lowering of blood glucose, normalization of glucose tolerance and insulin levels in islets, but surprisingly, also attenuated weight gain and adipogenicity despite comparable food intake. Histological examination of kidneys showed that saline control mice had severe inflammation, glomerular hypertrophy, and mesangial expansion, which were all attenuated in the IL233 treated mice. The protection correlated with greater accumulation of Tregs, group 2 innate lymphoid cells (ILC2), alternately activated macrophages and eosinophils in the adipose tissue, along with a skewing toward T-helper 2 responses. Thus, the novel IL233 cytokine bears therapeutic potential as it protects genetically obese mice from T2DN by regulating multiple contributors to pathogenesis.

**Short Description:** A novel bifunctional cytokine IL233, bearing IL-2 and IL-33 activities reverses inflammation and protects from type-2 diabetic nephropathy through promoting T-regulatory cells and type 2 immune response.

## Introduction

The alarmingly high incidence of obesity leading to diabetes and diabetic nephropathy (DN) is the largest contributor to chronic kidney disease and end-stage renal disease (ESRD) in the modern world (Collins et al., 2012). The current treatment strategies including better glycemic control and inhibitors of the renin-angiotensin system, are unable to completely restrict the DN-associated ESRD (Moreno et al., 2018). A growing collection of evidence points to inflammation as a key factor in the pathogenesis of DN (Pichler et al., 2017). Cellular metabolism and hemodynamic changes within the kidney trigger the activation of inflammatory pathways, causing functional and structural renal injury (Wada and Makino, 2013). Therefore, there is a potential in targeting the therapies to improve glycemic and hemodynamic control, as well as to reduce the inflammation and prevent renal damage. Recent studies show protective role of the anti-inflammatory T-regulatory cells (Tregs) in DN (Eller et al., 2011). Micro- and macro-albuminuria in diabetic patients had a significant inverse correlation with the levels of Tregs (Xu et al., 2009). Similarly, antibody-mediated depletion of Tregs worsened the proteinuria in diabetic (*db/db*) mice, while Treg-supplementation was protective (Eller et al., 2011).

Recent clinical studies show that an imbalance in the ratios of Tregs and Th2 cells to Th1 and Th17 cells contribute to overt DN in T2D patients and regulates the progression of T2D itself (Wu et al., 2010; Zeng et al., 2012). A therapeutic agent that can promote Tregs and Th2 cells, and restore the balance will be beneficial in blocking inflammation in DN and ESRD. We have identified the cooperativity of the signaling pathways of interleukin (IL)-2 and IL-33 (Stremska et al., 2017; Sharma and Kinsey, 2018). The role of IL-2 in promoting Tregs and immune-tolerance is well established (Fontenot et al., 2005; Bayer et al., 2007). IL-33 is an alarmin and was initially perceived as a proinflammatory cytokine (Liew, 2012). However, recent studies have demonstrated its role in Treg-biology and suppression of sterile inflammation (Peine et al., 2016). We hypothesized that owing to the expression of both IL-2 and IL-33 receptors, their cooperativity will promote the homeostasis of Tregs, Th2, and ILC2 and inhibit inflammation. To this effect, we generated a novel hybrid cytokine (IL233) bearing the activities of IL-2 and IL-33 in a single molecule, with a perceived potential to suppress multiple pathways of inflammation (Stremska et al., 2017). Indeed, IL233 outperformed IL-2 and IL-33 given either alone or in combination and protected mice from ischemic as well as nephrotoxic renal injury (Stremska et al., 2017). In the current study, we investigated the hypothesis that a therapy with the IL233 hybrid cytokine can be utilized to suppress inflammation associated with DN to prevent the progression to ESRD in a mouse model of obesity-linked DN.

## Materials and Methods

### Animal Model

All experimental procedures with animals were carried out in accordance with NIH Guide for the Care and Use of Laboratory Animals and approved by University of Virginia Animal Care and Use Committee. Male BTBR.Cg-*Lep^ob/ob^* (*Ob*) and BTBR.Cg-*Lep^ob/+^* (*Het*) mice were purchased from Jackson Laboratory (Bar Harbor, ME, United States). Mice were housed in conventional specific pathogen free facility on shredded paper bedding and were fed standard chow diet. The mice were injected with IL233 in 200 μl saline at 3.3 pmol/g/d intraperitoneally (i.p.) for 5 consecutive days starting either at 5 or 10 weeks of age. Control mice were injected with saline only. The recombinant cytokine was produced as described previously (Stremska et al., 2017). Mice were euthanized between the ages of 18–20 weeks when the majority of control animals had severe proteinuria.

### Functional Assessments

The renal function in mice was analyzed on regular intervals by measuring proteinuria using urinary MultiStix^TM^ (Bayer, United States) and microalbuminuria on collected urine using the microalbuminuria kit at the indicated time points (Albuwell M, Exocell Inc., United States) following manufacturer’s protocol. Urine was collected for 6 h from mice kept in sterilized metabolism cages with *ad libitum* access to food and water. Body weight and urine volume were recorded. Diabetes was monitored by measuring fasting blood glucose levels. Mice were fasted for 6 h in cages without any food and bedding, but with free access to water. Blood glucose was measured through tail-bleeds using electronic monitor and blood glucose test strips (ReliOn^TM^ Prime, United States). Intraperitoneal glucose tolerance (IPGTT) was measured as before (Meher et al., 2012). Briefly, mice were fasted for 12 h, fasting blood glucose levels were recorded and mice were injected intraperitoneally (i.p.) with 1 g/kg body weight of D-glucose. Blood glucose levels were documented at 10, 20, 30, 60, 90, and 120 min. To monitor food intake, mice were housed individually without bedding and the 24 h change in the weight of chow was recorded. Care was taken to include the chow crumbs that accumulated in the cages. The status of diabetes was also confirmed by measuring glycated proteins in the terminal plasma of the mice using the Mouse Glycated Serum Proteins (GSP) Assay Kit (Crystal Chem).

### Assessment of Kidneys and Pancreas

Kidneys and Pancreas were collected upon necropsy and transverse sections (5 μm) fixed in 10% buffered formalin were stained with hematoxylin and eosin (H&E) and evaluation for injury using light microscope (Axiovert 200, Zeiss, United States). Three random areas of each kidney section were used to assess the extent of kidney injury in a blinded manner. The score was assigned on a 0–4 scale with: 0-no kidney damage, 1-mild (0–20%), 2-moderate (20–40%), 3-high (40–60%), and 4-severe (>60%) presence of mononuclear infiltrates, tubular necrosis, cast formation, and glomerular hypertrophy. Mesangial expansion was characterized on Periodic acid–Schiff (PAS) stained sections and scored as 0-no mesangial expansion, 1-mild (0–20%), 2-moderate (20–40%), 3-high (40–60%), and 4-severe (>60%) of glomeruli with mesangial expansion.

Frozen sections (5 μm) of pancreas samples were stained with anti-insulin antibody (Abcam) and 4′,6-diamidino-2-phenylindole (DAPI; Thermo Fisher Scientific), mounted in VECTASHIELD (Vector) and analyzed for insulin production in the islets by immunofluorescence microscopy. At least 20 islets from different mice were marked and the insulin intensity normalized to islet area was measured using the MBF Bioscience Microscope System for Stereology and Tissue Morphology (Zeiss).

The presence of Tregs, Th1 and Th2 cells in the renal tissue was analyzed by semi-quantitative real-time PCR using the TaqMan^TM^ probes (Thermo Fisher Scientific) for *Fox3* (Mm00475162_m1), *Tbx21* (Mm00450960_m1), and *Gata3* (Mm00484683_m1), respectively, and CFX Real-Time PCR machine equipped with CFX Maestro Software (Bio-Rad).

### Flow Cytometry

Flow cytometry was performed as described previously (Stremska et al., 2017) using antibodies listed in Supplementary Table 1. Intracellular cytokines were analyzed on splenocytes *ex vivo* stimulated by phorbol 12-myristate 13-acetate (PMA) and Ionomycin in the presence of monensin as described earlier (Stremska et al., 2017). Data were acquired on a FACScan cytometer (BD Biosciences) with a 5-color upgrade (Cytek Development Inc.) and analyzed with FlowJo^TM^ software (FlowJo Inc.) using the gating strategies shown in Supplementary Figure 1.

### Statistics

Data are the summary of at least two independent experiments and a replicate of ≥6 mice for most experiments. Statistical analysis of comparison between the groups was carried out using one-way ANOVA, two-way ANOVA with repeated measures or unpaired *t*-test using GraphPad Prism^TM^ software. Results are expressed as the mean ± standard error with *n* ≥ 6. Results with *p* < 0.05 were considered significant; ^∗^*p* < 0.05, ^∗∗^*p* < 0.0, ^∗∗∗^*p* < 0.00, ^∗∗∗∗^*p* < 0.0001, NS *p* > 0.05.

## Results

### Treatment With IL233 Attenuated Hyperglycemia and Proteinuria

**FIGURE 1:**
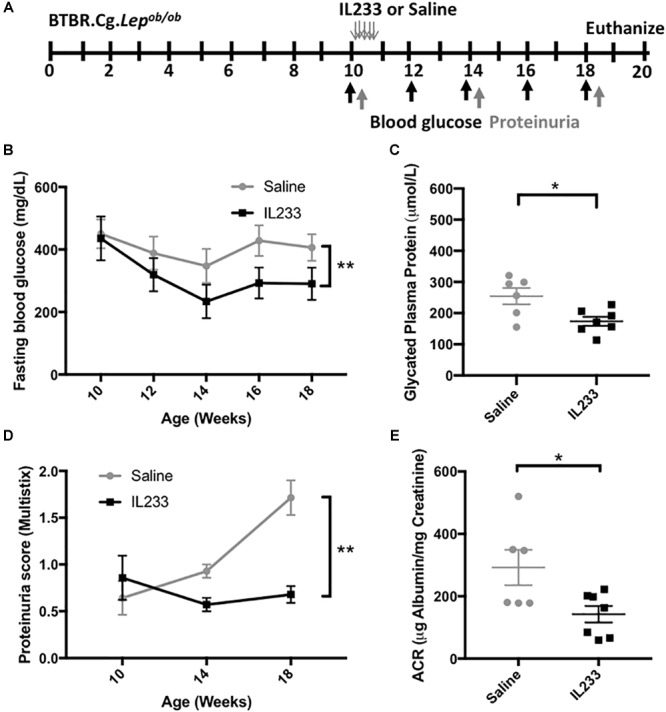
IL233 treatment protects genetically obese mice with established diabetes from hyperglycemia and diabetic nephropathy. **(A)** Experimental design; BTBR.Cg-*Lep^ob/ob^* mice (10 weeks old) were injected with saline or IL233 for 5 consecutive days and followed over time for diabetes and renal function. **(B)** Fasting blood glucose levels. **(C)** Glycated plasma proteins. **(D)** Proteinuria score using urinalysis Multistix^TM^. **(E)** Urinary albumin creatinine ratio (ACR) upon necropsy. Data compiled from two independent experiments. Mean ± SEM is shown (*n* = 7) at the beginning of the experiment. One saline control mouse died before the end of the experiment; Symbols represent individual mice. ^∗^*p* < 0.05; ^∗∗^*p* < 0.01; by two-way ANOVA with repeated measures **(B,D)** and non-parametric *t*-test **(C,E)**.

We used the BTBR.Cg-*Lep^ob/ob^* (*Ob*) mouse model, which develops spontaneous disease by 8 weeks of age (Hudkins et al., 2010), manifesting as rapid weight gain, early onset of T2D and measurable proteinuria. Based on our experience with other mouse models (Stremska et al., 2017), we injected 10 weeks old *Ob* mice (*n* = 7) i.p. with saline or 3.3 pmol/g body weight of IL233 daily for 5-days (Figure 1A). The mice were then monitored on regular intervals for blood glucose levels and proteinuria until the age of 18 weeks. Terminal proteinuria was measured by albumin creatinine ratio (ACR) at necropsy. At the beginning of the treatment all mice were severely diabetic, with fasting blood glucose >400 mg/dL. The saline-injected control mice remained hyperglycemic, whereas mice treated with IL233 rapidly showed a significant reduction over the following 4 weeks and stayed at the lower blood glucose levels thereafter (Figure 1B). There was a curious decline in the fasting blood glucose levels in both saline and IL233-treated mice, though this decline was statistically significant in the IL233 group, but not the saline group. It is possible that psychosocial stress due to handling and injection of these mice may have contributed to the declining fasting blood glucose initially, as has been described in the NOD mice (Durant et al., 1993). Upon necropsy, the saline treated (254 ± 26 μmol/L) *Ob* mice had significantly higher levels of glycated plasma proteins (Figure 1C) as compared to the IL233 treated group (173 ± 14 μmol/L). In addition, urine albumin showed a steady increase in saline control mice, whereas the IL233-treated mice had significantly lower proteinuria for the remainder of the experiment (Figure 1D). The ACR measurement confirmed that the IL233-treated mice (142 ± 27 μg/mg) had a significantly reduced albuminuria as compared to the control saline-treated (292 ± 57 μg/mg) mice (Figure 1E).

### Early IL233 Intervention Preserves Renal Structure and Function for Long-Term

**FIGURE 2:**
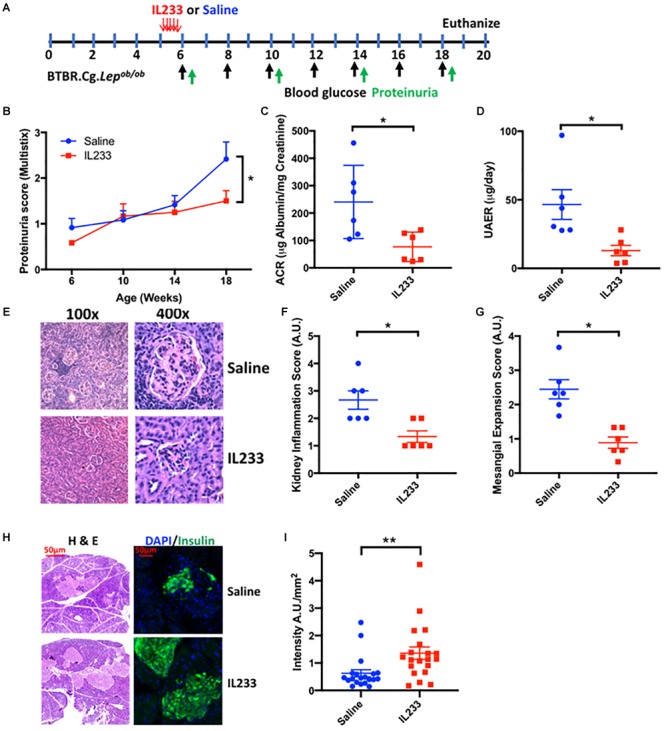
One time IL233 induction therapy of young mice offers long-term protection from loss of renal as well as pancreatic structure and function. **(A)** Experimental design; BTBR.Cg-*Lep^ob/ob^* mice (5 weeks old) were injected with saline or IL233 for 5 consecutive days and followed over time for proteinuria using urinalysis Multistix^TM^ **(B)**. **(C)** Urinary albumin creatinine ratio (ACR) and **(D)** Urinary albumin excretion rate (UAER) was measured at necropsy. **(E)** Representative kidney section using low and high power objectives. Quantification of kidney inflammation **(F)** and mesangial expansion **(G)** was performed according to the criteria mention in the Section “Materials and Methods.” **(H)** Representative hematoxylin and eosin (H&E) and immunofluorescence staining for insulin. **(I)** Quantification of insulin/mm^2^ area of islets. Data is compiled from two independent experiments. Mean ± SEM is shown (*n* = 6). Symbols represent individual mice; ^∗^*p* < 0.05; ^∗∗^*p* < 0.01; by non-parametric *t*-test and two-way ANOVA with repeated measures for **(B)**.

Prolonged obesity and diabetes correlate with higher incidence of chronic kidney disease. To test the hypothesis that early intervention with IL233 offers long-term protection in diabetic *Ob* mice, we injected (i.p.) 5 week old *Ob* mice with 3.3 pmol/g IL233 per day for 5 days and followed them until the age of 18 weeks (Figure 2A). Indeed, mice treated with IL233 showed a remarkable attenuation of proteinuria as measured with dipstick over time (Figure 2B) and a strong reduction in ACR (241 ± 55 μg/mg vs. 77 ± 22 μg/mg) and total albumin secretion UAER (47 ± 11 μg/d vs. 13 ± 3.8 μg/d) as compared to the saline control mice (Figure 2C,D). Meta-analysis of the ACR values (Supplementary Figure 2) indicated that early intervention resulted in significantly lower (77 ± 22 μg/mg) significant albuminuria compared to later treatment (142 ± 27 μg/mg). Histological analysis of kidneys further revealed severe tubulointerstitial and periglomerular inflammation along with marked glomerular hypertrophy and mesangial expansion in the saline control mice, all of which were significantly attenuated in the IL233 treated mice (Figure 2E–G). We also analyzed the pancreas of the saline and IL233 treated *Ob* mice at necropsy. In general, the H&E stained islets in the saline treated *Ob* mice appeared smaller and atrophic as compared to the IL233 treated mice (Figure 2H). We therefore stained the pancreatic islets for insulin and the data indicates that the saline-treated *Ob* mice expressed significantly less insulin/mm^2^ of islet area as compared to the IL233 treated mice (Figure 2I).

### Early Intervention With IL233 Restores Glucose Clearance and Inhibits Visceral Adiposity

**FIGURE 3:**
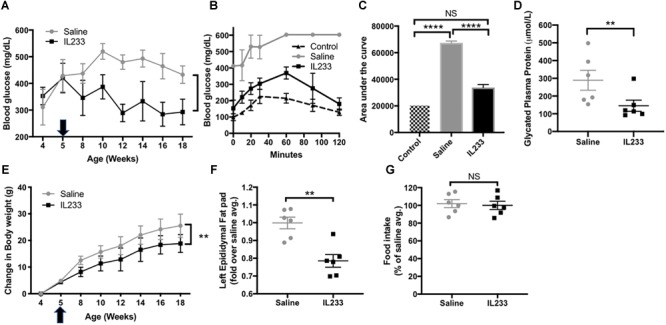
IL233 protects young genetically obese mice from diabetes and obesity. Young genetically obese mice were treated with saline or IL233 (arrow) as shown in Figure 2 and were followed over time for fasting blood glucose **(A)**. **(B)** Mice were tested for IPGTT around 15 weeks of age and compared with age and sex-matched lean non-diabetic (*Het*) control mice. **(C)** Area under the curve for IPGTT. **(D)** Glycated plasma protein levels. **(E)** Measurement of body weight. **(F)** Weight of left epididymal fat pad. **(G)** Analysis of 24 h food consumption. Data is compiled from two independent experiments. Mean ± SEM is shown (*n* = 6). Symbols represent individual mice; ^∗^*p* < 0.05; ^∗∗^*p* < 0.01; ^∗∗∗^*p* < 0.001; ^∗∗∗∗^*p* < 0.0001; NS *p* > 0.05 by non-parametric *t*-test **(D,F,G)**; two-way ANOVA with repeated measures for **(A,E)** and one-way ANOVA for **(C)**.

The importance of immunomodulation to increase Treg is recognized in type-1 diabetes (Yu et al., 2018), but whether the same mechanisms apply to obesity-linked diabetes is not known. We observed that an induction therapy in young *Ob* mice with daily injection of IL233 for 5-days was sufficient to inhibit progression of hyperglycemia and reverse the trend of increasing blood glucose levels as compared to the saline control mice (Figure 3A). We performed intraperitoneal glucose tolerance test (IPGTT) in these mice at 15 weeks of age, when the control mice were all morbidly obese with hyperglycemia and proteinuria, and compared them to age and sex-matched *Het* mice, which are lean and normoglycemic. The saline treated obese mice displayed a drastic impairment in glucose clearance and accumulated higher blood glucose over time, whereas IL233-treated mice efficiently cleared glucose and did not statistically differ from the normal *Het* control mice (Figure 3B,C). Further, the saline treated *Ob* mice (289 ± 56 μmol/L) had significantly higher levels of glycated plasma proteins compared to the IL233-treated group (145 ± 31 μmol/L), indicating sustained attenuation of diabetes by IL233 treatment (Figure 3D).

To our surprise treatment with the IL233 hybrid cytokine also attenuated the weight gain in these mice that are genetically susceptible to obesity (Figure 3E). In addition, the mass of visceral adipose tissue as measured in the left epididymal fat pad was significantly lower in the IL233-treated mice as compared to the saline controls (Figure 3F). Interestingly, there was no difference in the 24 h food consumption between the saline and IL233 treated groups (Figure 3G) indicating that a reduction in food consumption due to IL233-treatment did not contribute to lower body weight.

### Sustained Immunomodulation by IL233 May Contribute to Protection

**FIGURE 4:**
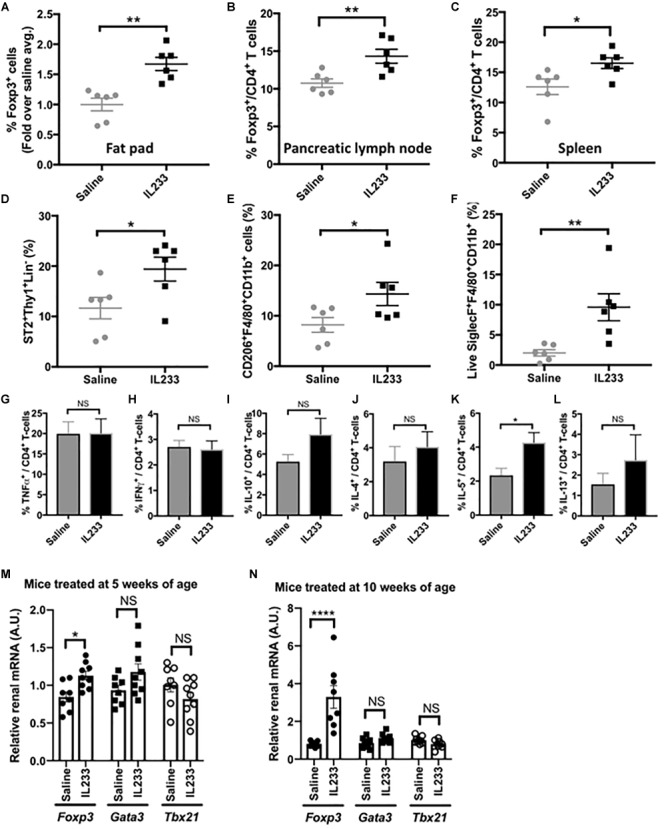
Analysis of immunomodulation by IL233 treatment. Flow cytometric analysis of Foxp3^+^ Tregs in epididymal fat pad **(A)**, Pancreatic lymph nodes **(B)** and Spleen **(C)**. Analysis of epididymal fat pad for ILC2 **(D)**, alternately activated macrophages **(E)**, and eosinophils **(F)** by flow cytometry. Intracellular cytokine analysis of PMA/Ionomycin stimulated splenocytes for TNFα **(G)**, IFNγ **(H)**, IL-10 **(I)**, IL-4 **(J)**, IL-5 **(K)**, and IL-13 **(L)**. Real-time PCR with TaqMan^TM^ probes for analysis of *Fox3, Gata3*, and *Tbx21* in the kidneys of mice treated either at 5 weeks **(M)** or 10 weeks of age **(N)**, Data is compiled from two independent experiments. Mean ± SEM is shown (*n* ≥ 6). Symbols represent individual mice; ^∗^*p* < 0.05; ^∗∗^*p* < 0.01; ^∗∗∗∗^*p* < 0.0001; NS *p* > 0.05 by non-parametric *t*-test.

We next investigated whether IL233 treatment of *Ob* mice induces sustained immunomodulation, which could contribute to protection from obesity, hyperglycemia and proteinuria. Treatment with 3.3 pmol/g of IL233 daily for 5 days, induced a rapid increase in the proportion of Tregs in circulation in both *Het* and *Ob* mice measured 8 days after the initiation of treatment, with sustained higher levels of Tregs on day 40, as compared to saline controls (Supplementary Figure 3A). Upon necropsy, it was found that the one-time induction therapy of 5 weeks old *Ob* mice with IL233 was sufficient to maintain a higher proportion of Tregs more than 13 weeks later in the adipose tissue, pancreatic lymph nodes, and spleen as compared to the saline controls (Figure 4A–C). Interestingly, the homozygous mutant *Ob* mice had significantly lower proportions of Tregs in the pancreatic lymph nodes as compared to age and sex matched *Het* mice, however, IL233 treatment restored the levels of Tregs in *Ob* mice to that of lean and normal *Het* mice (Supplementary Figure 3B). A similar trend was also observed in the spleens of *Het* and *Ob* mice (Supplementary Figure 3C) suggesting that systemic, as well as local levels of Tregs inversely correlate with obesity, diabetes, and renal dysfunction.

Since ILC2 also express the receptors for IL-2 and IL-33 and are known to populate adipose tissue, we analyzed their levels in our studies. Indeed, the proportion of ILC2 was significantly higher in the adipose tissue of IL233-treated mice (Figure 4D). In addition, IL233 treatment also increased the proportion of alternately activated macrophages (AAM; CD206^+^F4/80^+^CD11b^+^) and eosinophils (Siglec-F^+^F4/80^+^CD11b^+^) in the adipose tissue (Figure 4E,F). Although, there was no difference in the ability of splenic CD4^+^ T cells to produce TNFα or IFNγ (Figure 4G,H), which are associated with inflammation, we observed a trend for higher proportion of CD4^+^ T cells positive for IL-10, IL-4, and IL-13, all of which did not reach the threshold of statistical significance due to large biological variability (Figure 4I–K). However, IL-5 producing CD4^+^ T-cells in the spleen were significantly increased in mice treated with IL233 as compared to the saline controls (Figure 4L) and this correlated with a larger proportion of eosinophils in the adipose tissue (Figure 4F). Similar trend of skewing of the immune responses toward Th2 cytokines (IL-4, IL-5, IL-13) and immunomodulatory IL-10 was also observed in the pancreas draining lymph nodes (data not shown). Nevertheless, the ratio of Treg to TNFα or IFNγ producing T cells was significantly higher in the IL233-treated mice (Supplementary Figure 4).

We also analyzed the kidneys of the mice for the status of T-cell immune-modulation for its potential contribution to the protection from DN. As shown in Figure 4M,N, the IL233 treated mice had significantly higher expression of *Foxp3* in groups that were treated either at 5-weeks or 10-weeks of age. However, no significant difference in the expression of *Tbx21* (T-bet or Th1 cells) and *Gata3* (for Th2 cells) was observed in the kidneys of saline and IL233-treated *Ob* mice.

## Discussion

Diabetic nephropathy (DN) is a chronic renal disease, which has become one of the leading causes of ESRD and mortality globally (Collins et al., 2012). Although DN also occurs in association with autoimmune type-1 diabetes, obesity and obesity-linked diabetes is the major contributor to DN, and is associated with more than 60% cases of ESRD (Wada and Makino, 2013). Hyperglycemia and the downstream factors that are believed to drive pathogenesis include: generation of advanced glycation end products (AGE), protein kinase C-mediated generation of reactive oxygen species (ROS) or mechanical stress/shear injury due to renal hyperfiltration (Wada and Makino, 2013; Pichler et al., 2017), all of which can induce inflammation that plays a major role in the etiopathogenesis of DN.

Current treatment regimen for DN is directed toward optimizing glycemic control and lowering cardiovascular risk. However, recent studies have shown that most successful treatment strategies also contain immune-targeting properties (Moreno et al., 2018). Data from both animal and clinical studies have demonstrated a positive correlation of increase in inflammatory mediators with the pathogenesis (Pichler et al., 2017). Greater expression of chemotactic proteins and adhesion molecules in the kidneys, and a related accumulation of pro-inflammatory M1 macrophages has been reported in DN (Chow et al., 2004). Genetic ablation of macrophage migration associated molecules such as CCR2 or its ligand MCP1 attenuated diabetic kidney disease (Chow et al., 2007). Depletion of macrophages or inhibiting the survival of monocyte-derived macrophages was also shown to be protective in DN (Lim et al., 2009). Although, no direct role of T-cells has been established in the pathogenesis, higher numbers of circulating T-cells have been reported with heightened severity of DN (Bending et al., 1988; Moon et al., 2012). Accordingly, the T and B-cell deficient Rag1-deficient mice were protected from diabetic kidney disease as compared to Rag1-sufficient mice (Lim et al., 2010).

A higher ratio of Tregs to Th1 or Th17 cells was found to be protective against T2D and its downstream vascular complications (Zeng et al., 2012). Further, the ratio of Treg to Th1 or Th17 was much reduced in T2D patients as compared to healthy controls and was even more pronounced in patients with microvascular complications. In diabetic patients with overt nephropathy, the ratio of the Th1 to Th2 cytokines was drastically altered and Th1 cytokines were detectable in the urine of diabetic patients with overt nephropathy (Wu et al., 2010). Our data shows that in the BTBR*^ob/ob^* mouse model, the homozygous *Ob* mice had fewer Tregs than *Het* mice and IL233 treatment restored these levels, indicating a correlative role of Tregs with diabetes and DN (Supplementary Figure 3). Compared to the control mice, treatment with IL233 showed higher Foxp3 expression in the kidneys with no significant difference in *Tbx21* or *Gata3* levels. Systemic low-grade inflammation may be a contributing factor for the lower levels of Tregs in obesity, however, it is interesting to note that leptin has been shown to induce proinflammatory cytokines and inhibit Tregs in leptin/leptin receptor – sufficient mice fed high-fat diet (Matarese et al., 2010; Procaccini et al., 2010). Yet, the leptin-deficient BTBR*^ob/ob^* mice had heightened inflammation and reduced Treg proportions, indicating that factors other than leptin pathway may contributes to lower Tregs. This may be dictated by heightened innate immune cytokines in response to advanced glycation end-products, activation of inflammasomes, metabolic stress and TLR signaling (Akasheh et al., 2013; Gonzalez et al., 2013; Wada and Makino, 2016). Indeed, in our study IL233 treatment lowered inflammation, reduced hyperglycemia, and glycation index, whether administered either before or after the onset of diabetes and microalbuminuria.

Interestingly, Tregs have been shown to favor oxidative phosphorylation and fatty acid oxidation (Michalek et al., 2011; Priyadharshini et al., 2018) for metabolism, yet the hyperlipidemic animals in our current study and T2D patients (Wagner et al., 2013) had reduced Treg levels. This raises several important conundrums that need to be addressed experimentally in future studies. In animal studies, depletion of Tregs with anti-CD25 antibody accelerated DN in *db/db* mice and adoptive transfer of Tregs attenuated proteinuria and glomerular hypertrophy (Eller et al., 2011). Thus, Tregs offer an attractive approach for treatment of DN. However, generating and use of *ex vivo* expanded autologous or allogeneic Tregs has technical and biological limitations (Trzonkowski et al., 2015). Therefore, strategies that enhance endogenous Tregs are likely to be beneficial for therapy of DN.

Recently, we demonstrated a role for IL-33 in promoting Treg responses, such that IL-2 and IL-33 can synergize to increase Tregs and protect from experimental acute kidney injury (Stremska et al., 2017). Accordingly, we designed a hybrid cytokine (termed IL233) bearing the activities of IL-2 and IL-33 in a single molecule, which outperformed the mixture of IL-2 and IL-33 at equimolar ratios (Stremska et al., 2017). As anticipated IL233 treatment enhanced Tregs rapidly and in a sustained manner in the *Ob* mice, which correlated with strong inhibition of diabetes and DN. IL233 treatment also increased the ratio of Tregs to TNFα and IFNγ (Th1) cells in the *Ob* mice (Supplementary Figure 4). Higher TNFα levels correlate with increased severity of renal injury in diabetic patients (Navarro et al., 2006) and anti-TNF treatment attenuated proteinuria in experimental studies (Moriwaki et al., 2007). Interestingly, in our other recently published studies in Adriamycin nephropathy, intervention with IL233, 2 week after the induction of injury not only inhibited inflammation and restored renal function, but we also observed an induction of a reparative program marked by high expression of renal progenitors in the kidneys of mice treated with IL233 (Sabapathy et al., 2019). This was accompanied by an increase in the Tregs and ILC2 in the kidneys. Further, administration of an antibody to CD25, which depleted both Tregs and ILC2, ameliorated the protective effects. It is possible that a similar reparative response may also contribute to the observed protection from DN in the *Ob* mice.

The role of Tregs in type 1 diabetes is well appreciated and clinical studies for Treg-therapy have proven beneficial (Yu et al., 2018). Considering that defects in IL-2 production contribute to T1D, low-dose IL-2 therapy has been used to enhance Tregs experimentally as well as clinically with mixed results (Dwyer et al., 2016). Another autoimmune ailment that can lead to ESRD is systemic lupus erythematosus (SLE), where defects in IL-2 production and Tregs have been shown to contribute to pathogenesis (Mizui and Tsokos, 2016), and IL-2-based approaches to enhance Tregs have been shown to be beneficial for SLE but not for lupus nephritis (He et al., 2016; von Spee-Mayer et al., 2016). The use of IL-2 based approaches have been investigated for acute renal diseases including ischemia reperfusion and nephrotoxic injury (Kim et al., 2013; Stremska et al., 2017). IL-33 is an alarmin cytokine expressed in almost all cell types that is released from damaged or dying cells (Liew, 2012). Although, IL-33 has been proposed as a marker of acute renal injury, IL-33 levels were found to be similar between CKD patients and healthy controls. Soluble ST2 (sST2) can act as a decoy receptor to neutralize IL-33 activity and its levels were found to be higher in the circulation of CKD patients (Bao et al., 2012). Another study found that type-2 diabetes patients had elevated levels of IL-33 in the blood with no correlation to microalbuminuria (Caner et al., 2014). IL-33 was also recently shown to promote the recruitment of ILC2 into the white adipose tissue and promote “beiging” (Brestoff et al., 2015). Recently, IL-33 was also used to enhance Tregs and ILC2 for protection from acute renal injuries (Cao et al., 2018). However, no studies have been conducted for using cytokine based enhancement of Tregs for type 2 diabetes and/or T2DN. Although, type-2 diabetes have been considered to be associated with metabolic syndrome, the ability of the islets to lose insulin production cannot be ruled out. As observed in our study, the islets had significantly reduced expression of insulin in the islets as compared to the IL233 treated mice (Figure 2), suggesting a possible role of Tregs and suppression of inflammation in preserving islet function in type 2 diabetes, in addition to preserving renal structure and function.

Several studies have shown the benefits of Th2 response in obesity and adipogenicity (Zhang et al., 2015). IL-33 was also demonstrated in the B6.*Lep^ob/ob^* mouse model to protect from metabolic syndrome, induce Th2 response and polarize macrophages to an M2 phenotype, but no data on Tregs was reported (Miller et al., 2010). In the same study mice lacking IL-33 receptor (ST2) had increased body weight, adipogenicity, impaired insulin secretion, and glucose regulation compared to WT controls upon feeding of high-fat diet. Foxp3^+^ Tregs have been identified in adipose tissue, where they express IL-10 and PPAR-γ (Cipolletta et al., 2012). Clinically, mixed results have been obtained for correlation of Treg-levels with obesity (Eller et al., 2011; Zeyda et al., 2011; Agabiti-Rosei et al., 2018). In another study, treatment with IL-25 induced weight loss and improved glucose tolerance, accompanied with greater accumulation ILC2 and NKT cells, eosinophils, and AAM in visceral adipose tissue (Hams et al., 2013). In the same study depletion of ILC2 in Rag1 KO mice increased weight gain and glucose intolerance, whereas adoptive transfer of ILC2 or NKT cells induced transient weight loss and stabilized glucose homeostasis in obese mice. In our study, we observed that IL233 treatment increased the accumulation of Tregs, ILC2, eosinophils and AAM in the adipose tissue of genetically obese mice. This was accompanied with a trend toward Th2 skewing and a significant increase in IL-5 production, all of which could contribute to protection from adipogenicity, restoration of glucose clearance and decreased inflammation in the adipose tissue.

It is important to note that IL233-mediated protection was far more robust when offered to younger mice, because early intervention resulted in significantly lower albuminuria compared to the late intervention (Supplementary Figure 2). If such approaches were to be adopted clinically, treatment should be targeted to early onset obesity. We also observed a robust and sustained increase of Tregs in the pancreatic lymph node as well as spleens of the mice treated IL233, which likely contributed to reduced systemic inflammation as observed with higher Treg/TNFα ratio than saline-treated controls (Supplementary Figure 4). Thus, the IL233 hybrid cytokine, which contains the activities of IL-2 and IL-33, promotes Tregs systemically, inhibits inflammation, restores glucose homeostasis, promotes recruitment of ILC2, eosinophils, and AAM in the adipose tissue to attenuate obesity, offers multi-level protection from obesity linked diabetic nephropathy. Combined with a possibility of induction of a reparative program in kidneys (Sabapathy et al., 2019), IL233 bears strong therapeutic potential.

## Ethics Statement

This study was carried out in accordance with NIH Guide for the Care and Use of Laboratory Animals and approved by University of Virginia Animal Care and Use Committee.

## Author Contributions

RS conceived the project and designed the experiments. VS, MS, SM, RC, and PS performed the experiments and analyzed the data. RS, VS, and MS wrote the manuscript.

## Conflict of Interest Statement

The authors declare that the research was conducted in the absence of any commercial or financial relationships that could be construed as a potential conflict of interest.
