# Supplementary material for: Novel Immunomodulatory Cytokine Regulates Inflammation, Diabetes, and Obesity to Protect From Diabetic Nephropathy
Source: Front Pharmacol. 2019 May 22;10:572. doi: 10.3389/fphar.2019.00572 (PMC6540785; doi:10.3389/fphar.2019.00572)

Supplementary Material

Novel Immunomodulatory Cytokine Regulates Inflammation, Diabetes and Obesity to Protect from Diabetic Nephropathy

Vikram Sabapathy[1#], Marta E. Stremska[2#], Saleh Mohammad[1#], Rebecca L. Corey[1], Poonam R. Sharma[3], Rahul Sharma[1]*

The data presented here supports the results and conclusions reported in the main manuscript.

Supplementary Table 1. Antibodies used for flow cytometry.

Antigen	Conjugate	Catalog No.	Company
Foxp3	Alexa 488	53-5773-82	eBioscience
ST2	PE	145304	BioLegend
CD4	PercpCy5.5	100434	BioLegend
CD25	APC	102012	BioLegend
TCRβ	APCeflour780	47-5961-82	eBioscience
CD90	FITC	553013	BD Biosciences
NK1.1	PercpCy5.5	108728	BioLegend
B220	APC/Fire 750	103260	BioLegend
CD3	APC	551163	BD Biosciences
CD4	APC-eflour780	47-0041-82	eBioscience
CD8a	Percp-eflour710	46-0081-80	eBioscience
IFNγ	FITC	505806	BioLegend
IL-4	PE	554435	BioLegend
TNFα	FITC	11-7321-82	eBioscience
IL-10	PE	17-7101-81	eBioscience
IL-5	APC	153405	BioLegend
IL-13	APC	47-7133-82	eBioscience
IL-17	APC	506915	BioLegend
CD11b	APC/Fire 750	101261	BioLegend
F4/80	FITC	123107	BioLegend
CD206	APC	141707	BioLegend
Siglec-F	PE	155505	BioLegend

Supplementary Figure 1: Gating strategy for flow cytometry analysis using FlowJo™ software (FlowJo Inc).

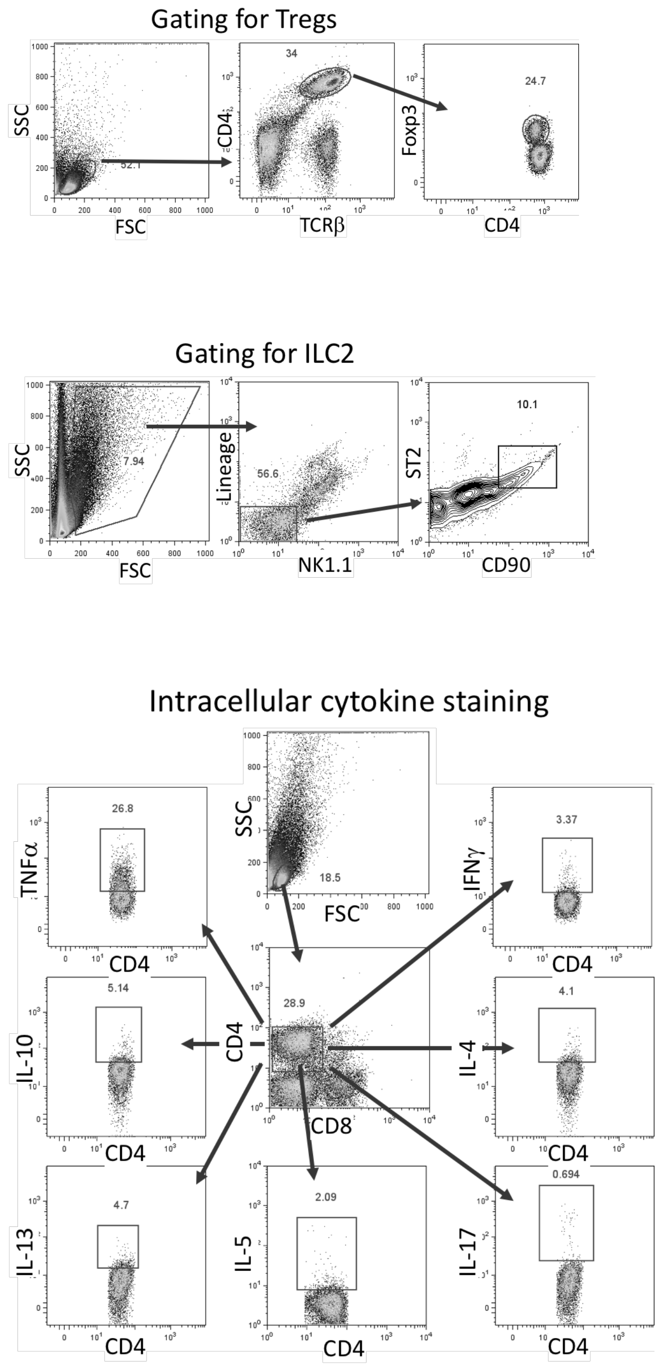

Supplementary Figure 2. Early intervention of BTBR.*Lep*$^{ob/ob}$ (*Ob*) mice at 5 weeks of age (Early) with IL233 show lower ACR values at necropsy compared to intervention at 10 weeks of age (Late). Mean ± SEM is shown (n=6); $p<0.05$ (*) by non-parametric T-test.

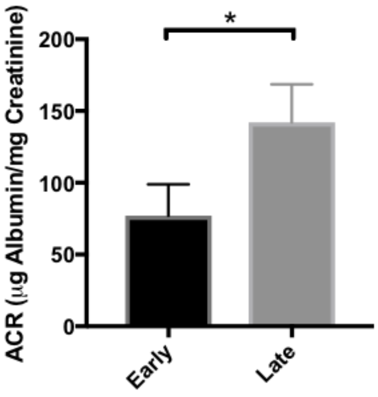

Supplementary Figure 3. BTBR.*Lep*$^{ob/ob}$ (*Ob*) and BTBR.*Lep*$^{ob/+}$ (*Het*) mice (5 weeks old) were treated with saline or 3.3pmol/g/day of IL233 (m233) for 5 consecutive days (arrows). **A.** The proportion of Foxp3^{+} Tregs was analyzed in the peripheral blood either before (d0) or after initiation of IL233 treatment (d8 and d40). The proportion of Foxp3^{+} Tregs was analyzed in **B.** Pancreatic lymph node (PLN) or **C.** Spleen (Spl) upon necropsy after 18 weeks of age. Mean ± SEM is shown (n=3); p<0.05 (*), p<0.01(**) by non-parametric T-test.

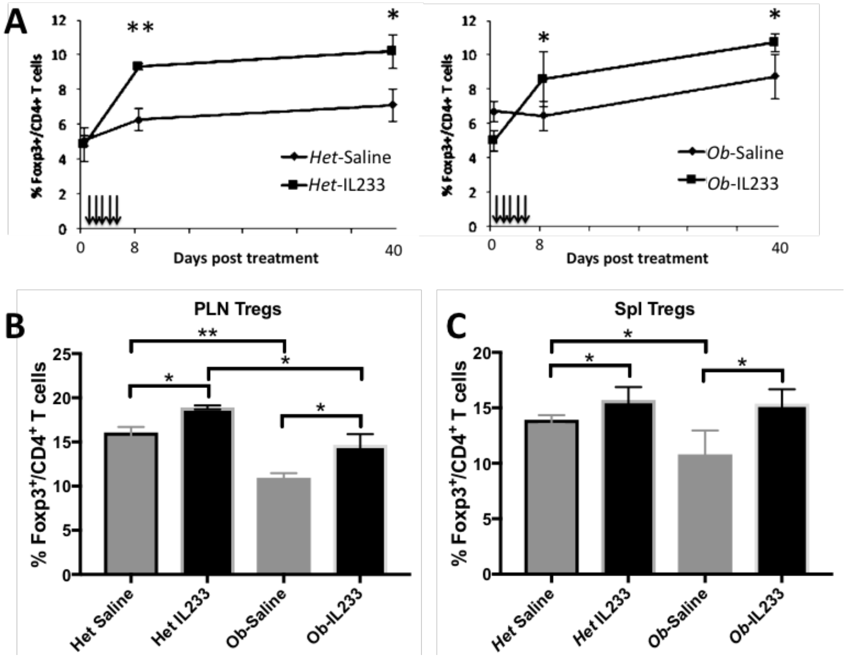

Supplementary Figure 4. BTBR.*Lep*$^{ob/ob}$ mice (5 weeks old) were treated with saline or 3.3pmol/g/day of IL233 for 5 consecutive days. The mice were euthanized after the age of 18 weeks and ratio of Tregs (Foxp3^{+}) cells to TNFα^{+} (**A**) or IFNγ^{+} **(B).** Mean±SEM is shown (n=6); $p<0.05$ (*), $p<0.01$(**).

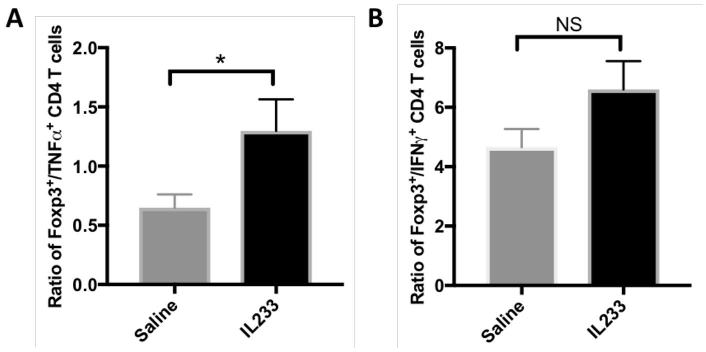

Supplement: Supplementary file 1 [file Data_Sheet_1.pdf]
